# Hereditary multiple exostoses of the ribs as an uncommon cause of pneumothorax: A case report

**DOI:** 10.1097/MD.0000000000011894

**Published:** 2018-08-21

**Authors:** Antoine Dumazet, Claire Launois, Sandra Dury, Frédéric Sailhan, Marco Alifano, Maxime Dewolf, François Lebargy, Gaëtan Deslee, Jeanne-Marie Perotin

**Affiliations:** a Department of Respiratory Diseases, University Hospital, Reims, France; b EA 4683, Laboratoire D’immunologie et de Biotechnologies, UFR de Pharmacie, Reims; c Department of Orthopedic Surgery, Cochin Hospital, APHP, Paris Descartes University; d Department of Thoracic Surgery, Cochin hospital, APHP, Paris Descartes University, Paris; e INSERM UMRS 1250, University Hospital, Reims, France.

**Keywords:** costal exostosis, hereditary multiple exostoses, pneumothorax, VATS

## Abstract

**Rationale:**

Hereditary multiple exostoses (HME) is a genetic musculoskeletal condition causing multiple exostoses. Rib location of exostosis can be complicated by thoracic injuries.

**Patient concerns and diagnoses:**

We report a case of pneumothorax in a 32-year-old man with a partial left-sided pneumothorax caused by an exostosis of the fourth and fifth ribs.

**Interventions and outcomes:**

Clinical and radiological presentations allowed a conservative management. A video-assisted thoracoscopic surgery was performed a few weeks later to avoid any recurrence.

**Lessons:**

Rib exostosis represents an unusual cause of pneumothorax. Any local modification of symptoms or size of the exostosis should lead to investigations in regard to chondrosarcoma transformation.

## 1 Introduction

Hereditary multiple exostoses (HME) is a genetic musculoskeletal condition with an autosomal dominant inheritance and a variable penetrance, involving *EXT1* and *EXT2* genes.^[1]^ HME is defined by the presence of at least two exostoses (or osteochondromas) of the juxta epiphyseal region of long bones. HME incidence is approximately 1:50000 in general population.^[2]^ The most frequent localizations of exostoses are around the knees and proximal humerus.^[3]^ Ribs exostoses are usually asymptomatic but can occasionally be associated with pleural, diaphragm or pericardial injuries. ^[4–6]^ We report a case of pneumothorax caused by costal exostosis.

## 2 Case report

**Figure 1:**
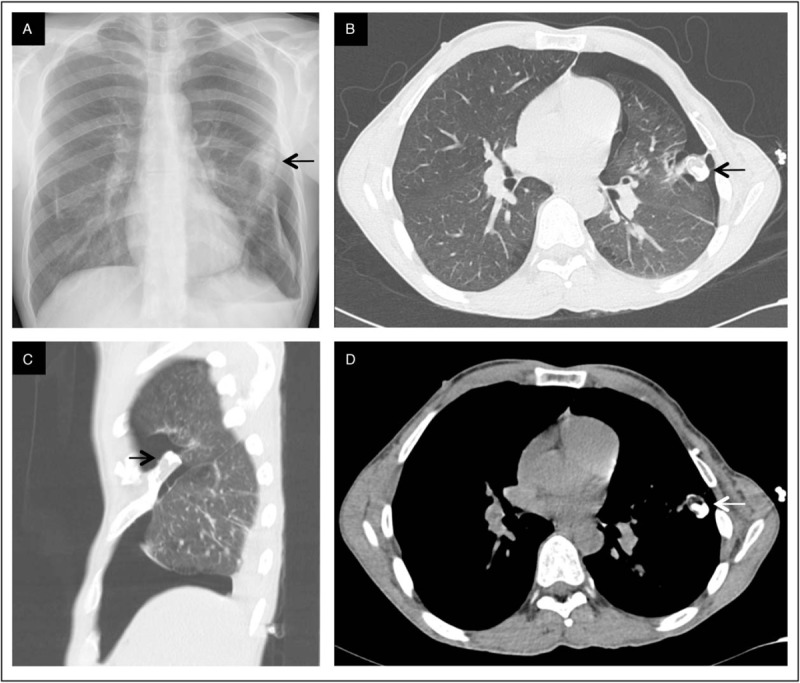
A, Chest X-ray showing left pneumothorax. B–D, Chest CT scan showing left pneumothorax, peripheral emphysema and rib exostosis.

A 32-year-old man was admitted for a spontaneous oppressive left side chest pain with a left arm irradiation for 2 days. He had a history of HME diagnosed in the childhood, with multiple leg exostosis resections and a leg-length inequalities correction. No genetic testing was available. He was a tobacco and cannabis smoker (13-pack-years). At admission, clinical exam did not reveal any sign of acute respiratory failure but a slight decrease in breath sounds in the left lung. Blood pressure was 130/80 mmHg, cardiac rate: 62 per minute, Sa02: 98%. Standard blood analysis and ECG were normal. A chest X-ray identified a left pneumothorax extending on axillary line and 2 dense opacities, 1 is located near the left fifth rib and the other being located near the right sixth rib (Fig. 1A). A chest computed tomography (CT) was performed and confirmed the left side pneumothorax and multiple costal exostoses (Fig. 1B–D). One exostosis was developed from the anterior arch of the left fifth rib with an intra-thoracic involvement and had a contact with the pneumothorax. Furthermore, CT-scan revealed bilateral paraseptal emphysema with an apical predominance.

Given clinical and radiological presentations, a conservative management was first proposed, resulting in a progressive and spontaneous improvement. The patient was discharged from hospital after 2 days management. Chest X-ray performed 2 weeks later exhibited complete resolution of the pneumothorax. Pulmonary function tests identified: forced expiratory volume in the first second (FEV_1_) 93% of predicted value, FEV_1_/forced vital capacity (FVC) 92%, RV 179% pred. The Alpha-1-antitrypsin level was normal.

**Figure 2:**
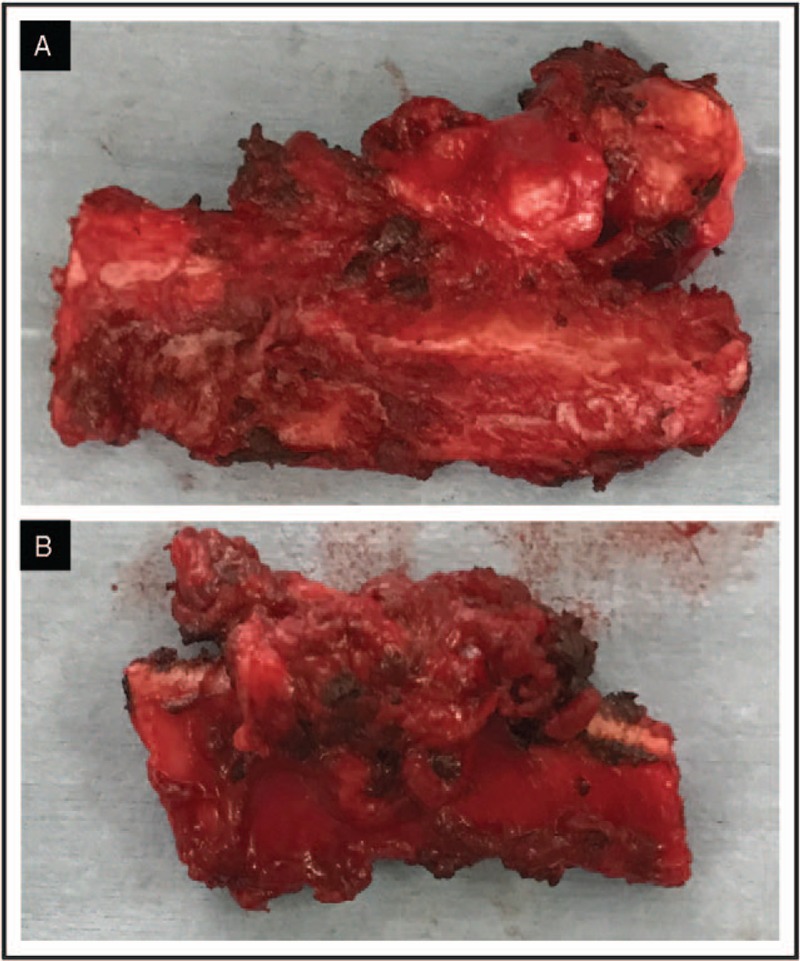
A, B, Surgical resection of rib exostoses.

Several weeks after this event, a surgical management of rib exostoses was proposed in order to prevent any pneumothorax recurrence. Surgery was performed by left-sided video-assisted thoracoscopy (VATS) and revealed exostoses of the left-sided fourth and fifth ribs with tight pulmonary adherences. A partial resection of the left-sided fourth and fifth ribs exhibiting intrathoracic exostosis lesions as well as a resection of 2 small emphysematous bullae were performed (Fig. 2). Due to double exostoses withdrawal, an early pulmonary hernia occurs and was taken care with a Vicryl plate to filling the anterior parietal defect.

Histological examination demonstrated emphysematous bullae and exostosis of the fourth and fifth ribs, with no sign of malignant transformation.

Written informed consent was obtained from the patient for publication of this case report.

## 3 Discussion

HME is a rare genetic musculoskeletal disease characterized by exostoses of long bones usually appearing and extending in the first decade of life with no extension after puberty. The number of exostoses is variable and more than 20 exostoses can occur in a patient. ^[1]^ Exostoses are mostly located around the knees and proximal humerus, usually sparing facial bones. Exostoses are usually asymptomatic but can induce different symptoms depending on exostoses localization, including pain, neurovascular compression, fractures or inequality in limb-length, as occurred in our case. Exostoses can also evolve with a chondrosarcoma transformation (0.5%– 5% of patients),^[7]^ which can be revealed by an increase in pain or size of the exostoses. Such symptoms should be evaluated by magnetic resonance imaging (MRI) and a removal of exostosis should be discussed.

Ribs exostoses are described between 35% and 44% of cases, depending on genotype (*EXT2* or *EXT1* respectively), and are usually asymptomatic.^[3]^ However, rib exostosis can occasionally be associated with hemothorax,^[4]^ pneumothorax,^[6]^ diaphragm or pericardial injuries. All 7 previously reported cases of pneumothorax^[5,6,8–12]^ occurred in young patients (12–36 years) and required surgical removal of the affected rib. We report here the second case of pneumothorax associated with a rib exostosis with a spontaneous improvement. The first was described by Assefa et al in a 15-year-old boy with a mild left apical pneumothorax. The surgical procedure in our case was performed several months after pneumothorax recovery in order to avoid any recurrence. Local recurrence rate is very low, less than 2%, especially after a complete excision and puberty.^[13]^

In our patient, the pneumothorax may have been induced by the close contact between the fifth rib exostosis and the pleura, as well as by paraseptal emphysema. No association between HME and emphysema has been previously described. In our case, emphysema could be related to tobacco and cannabis use. Alpha-1-antitrypsin level was normal.

HME physiopathology involves *EXT1* and *EXT2* genes, located respectively on chromosome 8 and 11. ^[1]^*EXT1* mutation and male gender are associated to a more severe HME phenotype with a greater degree of functional limitation and deformity.^[3]^

*EXT1* and *EXT2* genes are tumor suppressor genes coding for exostosins 1 and 2, two glycosyl-transferases required for the biosynthesis of heparan sulfate. Exostosins 1 and 2 are ubiquitous ^[14]^ predominantly present in the lung and are thought to be involved in vascular development and angiogenesis in endothelial cells of adult lung.^[15]^ A role of *EXT1* in asthma has also been suggested by Nonaka et al in an *EXT1* knockout mouse model of asthma.^[16]^ However, the role of *EXT* genes and exostosins in lung development and pathology remains to be elucidated.

In conclusion, rib exostosis represents an unusual cause of pneumothorax. Although a spontaneous improvement of pneumothorax can occur, rib exostosis removal can be performed to avoid any recurrence. Any local modification of symptoms or size of the exostosis should lead to investigations in regard to chondrosarcoma transformation

## Author contributions

**Conceptualization:** Antoine Dumazet.

**Writing – original draft:** Antoine Dumazet, Jeanne-Marie Perotin.

**Writing – review & editing:** Antoine Dumazet, Claire Launois, Sandra Dury, Frédéric Sailhan, Marco Alifano, Maxime Dewolf, François Lebargy, Gaëtan Deslee.
